# Pain Without Words: Emoji-Based Assessment of Acute Pain in the Emergency Department—Clinical and Medico-Legal Impact

**DOI:** 10.3390/jcm15176666

**Published:** 2026-08-28

**Authors:** Bruno Cirillo, Simona Meneghini, Roberto Cirocchi, Gabriele Napoletano, Aniello Maiese, Andrea Mingoli, Giovanna Sgarzini, Giacomo Bonito, Paola Frati, Matteo Matteucci, Enrico Marinelli, Lina De Paola

**Affiliations:** 1 Department of Surgery, Sapienza University of Rome, 00185 Rome, Italy; bruno.cirillo@uniroma1.it (B.C.); simona.meneghini@uniroma1.it (S.M.); andrea.mingoli@uniroma1.it (A.M.); 2 Department of Surgery, General Surgery, University of Perugia, 06123 Perugia, Italy; roberto.cirocchi@unipg.it; 3 Department of Anatomical, Histological, Forensic and Orthopedic Sciences, Sapienza University of Rome, 00185 Rome, Italy; gabriele.napoletano@uniroma1.it (G.N.); aniello.maiese@uniroma1.it (A.M.); paola.frati@uniroma1.it (P.F.); 4 Department of Risk Management and Legal Medicine, San Giovanni Hospital, 00184 Rome, Italy; 5 Department of Emergency Radiology, Sapienza University of Rome, 00185 Rome, Italy; giacomo.bonito@uniroma1.it; 6 Department of Surgery, University of Milan, 20122 Milan, Italy; matteo.matteucci@unimi.it; 7 Department of Medico-Surgical Sciences and Biotechnologies, Sapienza University of Rome, 04100 Latina, Italy; enrico.marinelli@uniroma1.it

**Keywords:** pain, pain assessment, emoji scale, emergency department, language barriers, visual communication, ordinal agreement, medico-legal implications

## Abstract

**Background:** Pain is among the most frequent reasons for seeking medical care and presenting to the Emergency Department (ED). In Italy, Law No. 38/2010 establishes the right to pain therapy and requires pain intensity and its evolution during treatment to be assessed and documented. This process may be challenging in patients with language barriers because conventional verbal or numerical instruments require a degree of linguistic or numerical comprehension. Emoji-based visual tools may provide an additional means of communicating pain intensity in linguistically diverse clinical settings. **Methods:** We conducted a single-center, prospective, observational pilot study involving adult patients presenting to the ED with acute pain and a clinically relevant language barrier. Pain was assessed before and after analgesic treatment using a six-category Emoji-Based Pain Scale, scored 0, 2, 4, 6, 8, and 10, and the 11-point Numeric Rating Scale (NRS). Feasibility, independent completion, monotonic association, and agreement between the instruments were evaluated. Spearman’s rank correlation, exploratory Bland–Altman analysis, and quadratic-weighted Cohen’s kappa were calculated separately before and after treatment. A post hoc ancillary rank-ordering exercise was subsequently conducted in 50 adult volunteers using the same unlabelled and randomly shuffled emoji cards. **Results:** Eighty-seven ED patients were enrolled. The Emoji-Based Pain Scale was completed independently by 78 patients (89.7%), compared with 61 patients (70.1%) for the NRS. Mean pre-treatment scores were 4.90 ± 1.77 for the Emoji-Based Pain Scale and 5.15 ± 1.65 for the NRS; post-treatment scores were 2.16 ± 1.62 and 2.46 ± 1.73, respectively. Strong positive associations were observed before treatment (Spearman’s ρ = 0.860, *p* < 0.001) and after treatment (ρ = 0.805, *p* < 0.001). Mean paired differences were −0.25 points before treatment and −0.30 points after treatment. Quadratic-weighted kappa was 0.747 before treatment (95% bootstrap confidence interval [CI], 0.661–0.818) and 0.720 after treatment (95% bootstrap CI, 0.609–0.806). Exact categorical agreement was observed in 62.1% and 58.6% of assessments, respectively. In the ancillary exercise, all 50 volunteers reproduced the prespecified emoji sequence, with no inversion of the two upper anchors. **Conclusions:** The Emoji-Based Pain Scale was feasible and more frequently completed without assistance than the NRS in this selected population of adult ED patients with language barriers. Strong monotonic association, good ordinal agreement, and limited mean differences were observed between the instruments. These findings support further evaluation of the scale but do not establish formal validity, reliability, or interchangeability. Direct psychometric comparison with established visual pain scales remains necessary before routine clinical integration.

## 1. Introduction

Pain is among the most frequently reported symptoms and one of the leading reasons for seeking medical attention. The International Association for the Study of Pain defines pain as “an unpleasant sensory and emotional experience associated with, or resembling that associated with, actual or potential tissue damage” [1,2]. This definition emphasizes that pain is subjective and multidimensional and cannot be inferred solely from the presence or severity of tissue injury. Accurate pain assessment is central to clinical decision-making, therapeutic monitoring, and the protection of patient dignity. In Italy, Law No. 38 of 15 March 2010 established the right of citizens to access palliative care and pain therapy and introduced specific requirements concerning the assessment and documentation of pain in the medical record [3]. These obligations may be difficult to fulfil when patients cannot communicate effectively because of linguistic, educational, or numerical-literacy barriers. This problem is particularly relevant in the Emergency Department, where clinical decisions must often be made rapidly and patients may come from diverse linguistic and cultural backgrounds. Trauma also represents an important component of contemporary emergency-care activity, including injuries associated with emerging forms of urban mobility. Conventional pain scales remain essential clinical instruments, but their use may be hindered when the patient and clinician do not share a language or when the patient is unfamiliar with numerical rating systems. Visual-symbol-based tools may offer an additional communication pathway in such circumstances. Emojis are familiar digital pictograms used extensively in contemporary communication. Familiarity, however, should not be equated with universal or culturally invariant interpretation. Their meaning may differ according to individual experience, cultural context, and graphical rendering. Emoji-based pain instruments therefore require specific empirical evaluation rather than an assumption of universal comprehensibility. The aim of this pilot study was to assess the feasibility and usability of a six-category Emoji-Based Pain Scale in adult ED patients with language barriers and to explore its association and agreement with the NRS before and after analgesic treatment. The study was not designed to establish definitive psychometric validity or reliability. A post hoc ancillary rank-ordering exercise was subsequently undertaken to determine whether the selected emojis were spontaneously arranged according to the prespecified pain-intensity sequence when presented without labels, numbers, or positional cues.

### 1.1. Pain Assessment and the Italian Legal Framework

Pain has clinical, ethical, and legal relevance. Italian Law No. 38/2010 requires pain intensity and its evolution during treatment to be documented in the medical record using appropriate assessment instruments [3]. The legislation reflects a broader healthcare principle: avoidable suffering should be actively identified and treated rather than regarded as an inevitable consequence of disease. Pain management should be individualized and may include pharmacological, psychological, rehabilitative, interventional, or surgical strategies, depending on the underlying condition and clinical context [4,5]. Specific recommendations have also been developed for cancer-related pain [6]. Healthcare professionals therefore have a duty to make reasonable efforts to assess and treat pain even when conventional communication is impaired. Language discordance may interfere with symptom characterization, informed participation, timely analgesia, and documentation. Non-verbal visual instruments may contribute to overcoming part of this barrier, although they cannot replace professional interpretation or cultural mediation when more complex clinical communication is required.

### 1.2. Conventional and Visual Pain Scales

**Figure 1:**
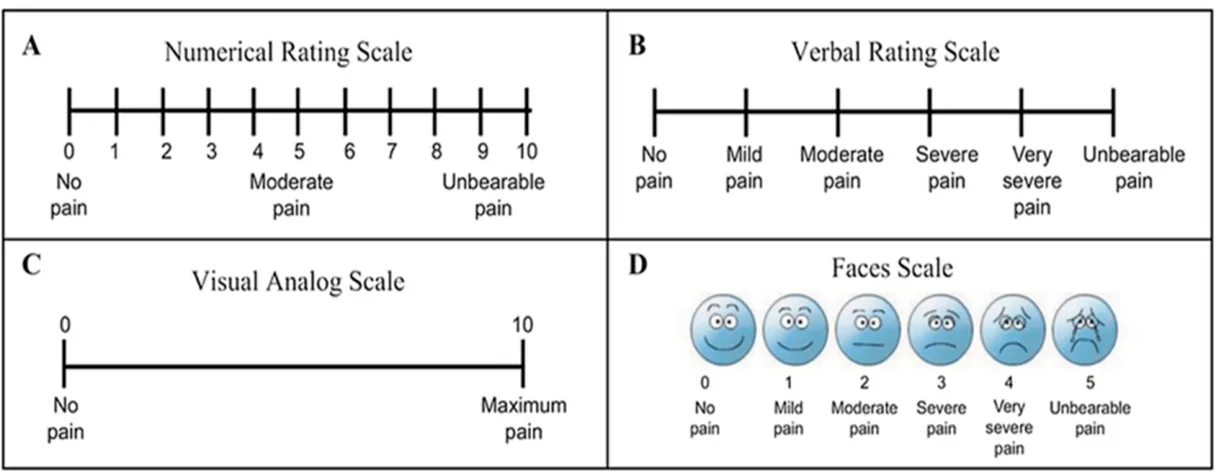
Overview of commonly used unidimensional pain-assessment scales. (**A**) Numerical Rating Scale; (**B**) Verbal Rating Scale; (**C**) Visual Analog Scale; (**D**) Wong-Baker Faces Scale.

Pain intensity is commonly measured using unidimensional instruments such as the Visual Analogue Scale (VAS), Verbal Rating Scale (VRS), and Numeric Rating Scale (NRS) [7,8] (Table 1) (Figure 1). The NRS generally asks patients to select an integer from 0, representing no pain, to 10, representing the worst imaginable painThe measurement properties and international applicability of these instruments have been examined in different adult populations [9,10]. Multidimensional instruments assess additional features such as pain quality, duration, emotional consequences, and interference with daily activities. These tools are particularly relevant to chronic pain but are often less practical in emergency settings. Although conventional scales are widely used, their performance depends partly on the patient’s ability to understand the response format. Numerical scales may be difficult for patients with low numeracy, limited education, cognitive impairment, or language discordance. Visual and face-based scales may therefore be useful in selected populations [11,12,13]. The Wong–Baker FACES Pain Rating Scale was initially developed for children and comprises six facial expressions associated with scores ranging from 0 to 10 [13]. Responsiveness may also differ between verbal and numerical pain-rating formats [12]. The visual structure of face-based scales has stimulated the development of related approaches in other populations. The present Emoji-Based Pain Scale inherited the general concept of a six-level facial progression but used a newly selected sequence of emojis rather than the empirically developed Wong–Baker faces [13,14,15,16].

### 1.3. Emojis and Their Potential Relevance to Healthcare

The term emoji derives from the Japanese words e, meaning picture, and moji, meaning character. Emojis were introduced in Japanese mobile communication during the late 1990s and subsequently became integrated into major digital platforms [17]. The Unicode Consortium assigns standardized code points and descriptive names to emojis, thereby enabling digital interoperability. Unicode standardization does not, however, ensure identical graphical appearance across operating systems, devices, or software vendors. The same code point may be rendered differently by different platforms [14]. Emojis have been investigated as tools for communicating pain and emotional states [15,18]. Their potential use in emergency and broader healthcare settings has also been explored [19,20]. Wider analyses have considered both the opportunities and the interpretative limitations of emoji-based medical communication [20,21]. He et al. compared an emoji-based visual analogue pain scale with an NRS and reported substantial agreement between the instruments in a single-hospital sample [15]. The interpretation and methodological implications of that study were subsequently discussed in published correspondence [16,17]. Li et al. later developed and evaluated an Emoji Faces Pain Scale on mobile devices in adult surgical patients, including direct comparison with established pain instruments [22,23,24,25]. These studies support the scientific plausibility of emoji-based pain communication while also demonstrating the importance of formal instrument development and validation. The present study differs by focusing specifically on adult ED patients with language barriers and by using a fixed, printed, six-category emoji sequence.

## 2. Materials and Methods

### 2.1. Study Design and Setting

This was a single-center, prospective, observational pilot study conducted between January 2022 and December 2024 in the Emergency Department of Policlinico Umberto I, Sapienza University of Rome, a tertiary-care university hospital. No intervention outside routine clinical practice was performed. Analgesic treatment was prescribed independently by the treating clinicians according to each patient’s clinical condition and was not determined by the study protocol.

### 2.2. Study Population and Recruitment

Potentially eligible patients were assessed consecutively during periods in which trained study personnel were available.

Patients were eligible if they

1. Were aged 18 years or older;
2. Presented to the ED with acute pain, irrespective of aetiology;
3. Were unable to communicate fluently in Italian and had a language barrier considered likely to interfere with routine clinical interaction; and
4. Could nevertheless establish sufficient communication through a vehicular language, primarily English or French, an available bilingual healthcare professional, or a cultural mediator.

Patients were excluded if they had cognitive impairment or altered mental status preventing reliable self-report, had received analgesic treatment before the initial study assessment, had severe visual impairment, had previously participated in the study, or declined participation. The exclusion of patients with cognitive impairment represented a methodological scope boundary. The study was designed to evaluate independent self-report and was not intended to assess proxy reporting or pain measurement in patients with impaired cognition. The ED case mix included patients with major trauma [26], altered consciousness, cognitive impairment, and other conditions that frequently precluded self-reported pain assessment. Moreover, the hospital catchment population included a relatively limited number of non-Italian-speaking residents. These factors contributed to the modest recruitment rate over the study period. A complete prospective screening log was not maintained. Consequently, the total number of potentially eligible patients and the number excluded for each reason could not be reconstructed reliably. Patients for whom no functional vehicular language or mediation support was available could not be enrolled. The study population therefore represented a comparatively more communicatively accessible subgroup within the broader population of patients with language barriers.

### 2.3. Communication and Informed Consent

Study objectives and procedures were explained in simple terms using a vehicular language, most commonly English or French, or with the assistance of a bilingual healthcare professional or cultural mediator. Informed consent was obtained from all participants in the presence of a witness, in accordance with the approved minimal-risk protocol. No patient was enrolled when sufficient understanding of the study procedure could not be established.

### 2.4. Pain-Assessment Instruments

The first instrument was a six-category Emoji-Based Pain Scale conceptually inspired by face-based pain scales. It consisted of the following fixed sequence:

- U+1F600, grinning face: score 0;
- U+1F610, neutral face: score 2;
- U+1F641, slightly frowning face: score 4;
- U+1F61F, worried face: score 6;
- U+1F623, persevering face: score 8;
- U+1F629, weary face: score 10.

The emojis were printed using a single fixed graphical rendering on laminated cards and displayed horizontally in ascending order during the clinical study. The use of a fixed printed rendering eliminated within-study variation attributable to different devices or operating systems [14]. The second instrument was the 11-point NRS, ranging from 0, no pain, to 10, worst imaginable pain. The NRS was selected as an established numerical comparator because of its widespread use and documented measurement properties in adult pain assessment [7,8]. Additional comparative studies have supported its clinical utility across different populations [9,10]. It was not regarded as an absolute gold standard. The Emoji-Based Pain Scale was administered first, followed immediately by the NRS. The sequence was standardized for all participants. Each instrument was explained separately, and patients were asked to reassess their pain independently. No feedback regarding the previous response was provided.

### 2.5. Clinical Assessments

Pain was assessed at two timepoints:

1. Before administration of analgesic treatment; and
2. After analgesic treatment delivered as part of routine clinical care.

**Figure 2:**
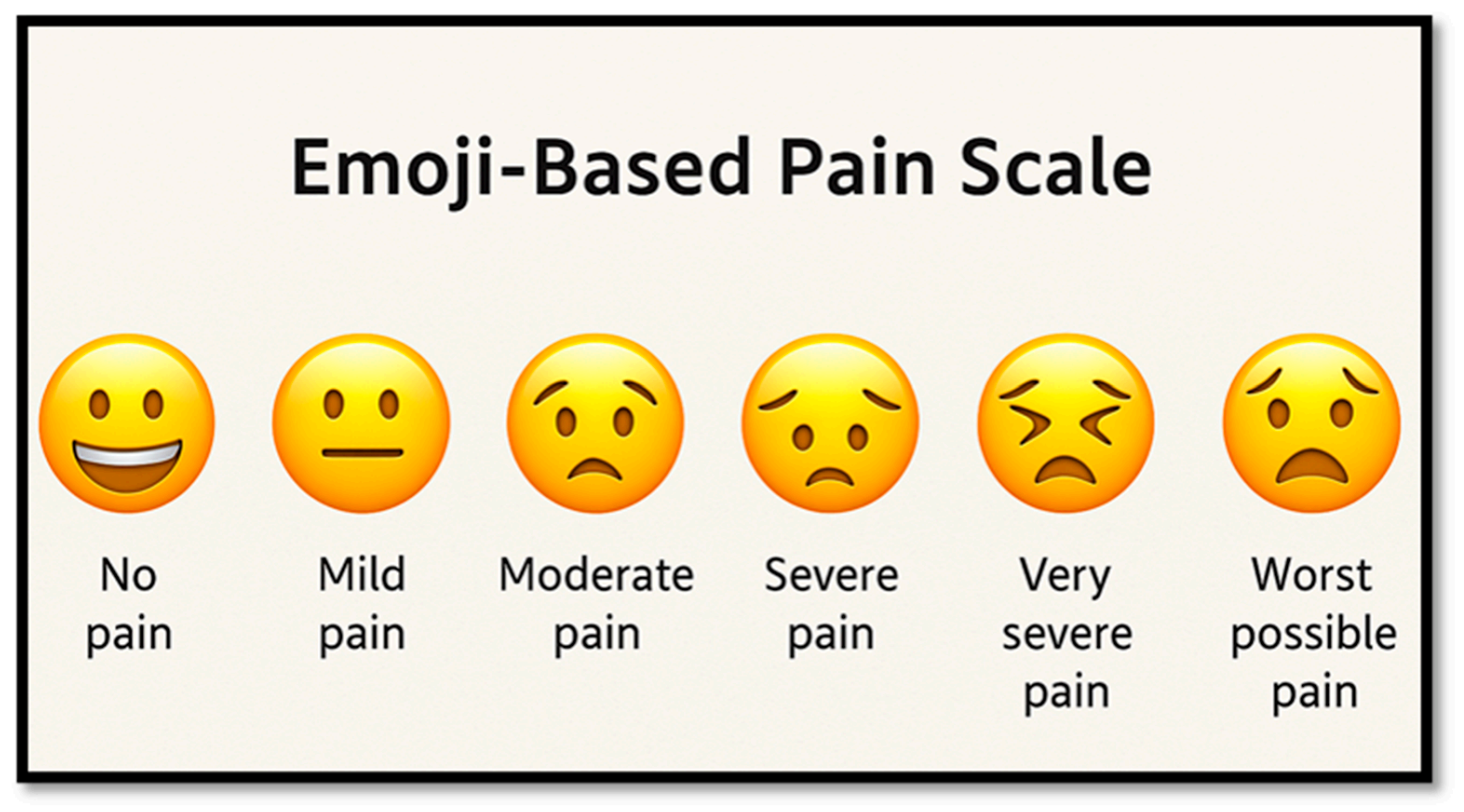
Emoji-Based Pain Scale. Visual scale composed of six emojis representing increasing levels of pain intensity, from “no pain” to “worst possible pain,” designed for use in patients with communication or language barriers.

At each timepoint, the Emoji-Based Pain Scale was administered first (Figure 2) and the NRS immediately afterwards. The investigators recorded whether each instrument was completed independently or whether brief clarification or assistance was required. These observations were used to evaluate feasibility and usability.

### 2.6. Ancillary Emoji Rank-Ordering Exercise

Following concerns regarding whether the selected glyphs conveyed an intrinsic pain-intensity gradient, a post hoc ancillary rank-ordering exercise was conducted in 50 adult volunteers who had not participated in the ED study and had not previously been shown the ordered scale. The same six emojis and the same fixed rendering used on the laminated clinical cards were presented as separate cards. Before each assessment, the cards were shuffled. No numerical values, pain descriptors, Unicode names, or other labels were shown.

Participants received the following standardized instruction:

“These six faces are shown in random order. Please arrange them from the face that, in your opinion, represents no pain to the face that represents the worst possible pain. Base your answer only on the images.”

No guidance, correction, or feedback was provided. The final sequence selected by each participant was recorded anonymously. The primary outcome was the proportion of participants reproducing the complete prespecified sequence. Secondary outcomes included inversion of the two upper anchors and overall interparticipant concordance. The ancillary exercise involved anonymous data collection, no clinical intervention, and no linkage to the original patient dataset. Participation was voluntary.

### 2.7. Statistical Analysis

Statistical analyses were performed using IBM SPSS Statistics for Windows, version 28.0 (IBM Corp., Armonk, NY, USA). Continuous variables were summarized as means and standard deviations or medians and interquartile ranges, as appropriate. Categorical variables were reported as frequencies and percentages. Because the Emoji-Based Pain Scale has six ordered categories and the data contained numerous tied observations, monotonic association with the NRS was evaluated using Spearman’s rank correlation coefficient. Agreement was explored using Bland–Altman analysis. For each paired assessment, the difference was calculated as the Emoji-Based Pain Scale score minus the NRS score. Mean paired differences and 95% limits of agreement were calculated separately for pre-treatment and post-treatment assessments. Although the Emoji-Based Pain Scale is ordinal, its six categories were assigned evenly spaced values of 0, 2, 4, 6, 8, and 10. These assigned scores were treated as an interval proxy solely for the exploratory Bland–Altman analysis. This analysis was therefore interpreted descriptively and not as definitive evidence of measurement equivalence.

Ordinal agreement was additionally assessed using quadratic-weighted Cohen’s kappa. For this analysis, NRS scores were collapsed into six prespecified categories corresponding to the Emoji-Based Pain Scale:

- NRS 0 was mapped to emoji score 0;
- NRS 1–2 to emoji score 2;
- NRS 3–4 to emoji score 4;
- NRS 5–6 to emoji score 6;
- NRS 7–8 to emoji score 8; and
- NRS 9–10 to emoji score 10.

Quadratic weights were selected to account for the ordered nature of the categories and to assign progressively greater importance to larger disagreements. Ninety-five per cent confidence intervals for weighted kappa were estimated using 10,000 bootstrap resamples. Exact categorical agreement was also reported. Pre-treatment and post-treatment observations were analysed separately to avoid treating repeated measurements from the same patient as independent observations. For the ancillary ordering exercise, exact agreement with the complete prespecified sequence and inversion of the two upper anchors were reported as frequencies and percentages. Overall concordance among participants was assessed using Kendall’s coefficient of concordance.

All statistical tests were two-sided, and *p* < 0.05 was considered statistically significant.

## 3. Results

### 3.1. Clinical Cohort and Instrument Usability

A total of 87 patients were enrolled between January 2022 and December 2024. Their mean age was 44 years, with a range of 19–76 years, and 53 patients were male (60.9%). Participants came from North Africa, sub-Saharan Africa, South Asia, Eastern Europe, and Southeast Asia. The most frequent clinical presentations were abdominal pain, trauma, and musculoskeletal injuries. The Emoji-Based Pain Scale was completed independently by 78 patients (89.7%), while 9 patients (10.3%) required brief clarification or assistance. The NRS was completed independently by 61 patients (70.1%), whereas 26 patients (29.9%) required clarification or assistance in using the numerical response format.

### 3.2. Pain Scores Before and After Treatment

Before treatment, the mean Emoji-Based Pain Scale score was 4.90 ± 1.77, compared with 5.15 ± 1.65 on the NRS. After treatment, mean scores decreased to 2.16 ± 1.62 on the Emoji-Based Pain Scale and 2.46 ± 1.73 on the NRS, as shown in Table 2. No patient selected the maximum emoji category corresponding to score 10.

**Figure 3:**
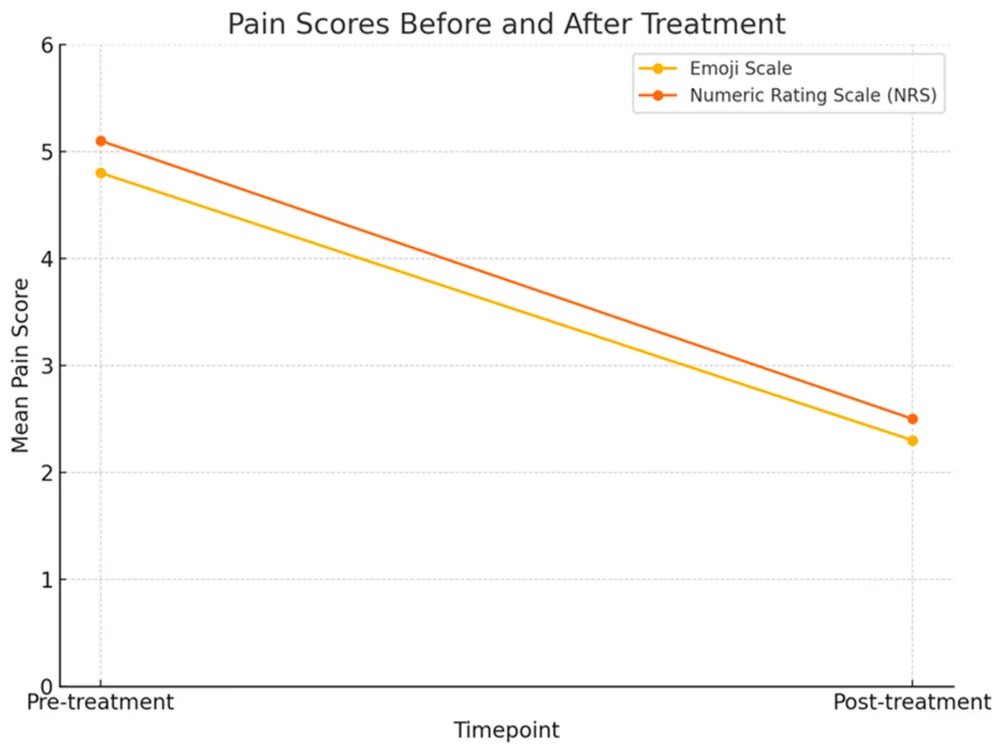
Mean pain scores before and after treatment using Emoji and NRS Scales. Mean scores on the Emoji Scale and NRS both decreased significantly post-treatment.

The parallel decrease observed with both instruments suggests that the Emoji-Based Pain Scale may reflect changes in self-reported pain following treatment. This finding should be interpreted as preliminary evidence of potential responsiveness rather than proof of formal responsiveness or validity (Figure 3).

### 3.3. Association and Agreement

**Figure 4:**
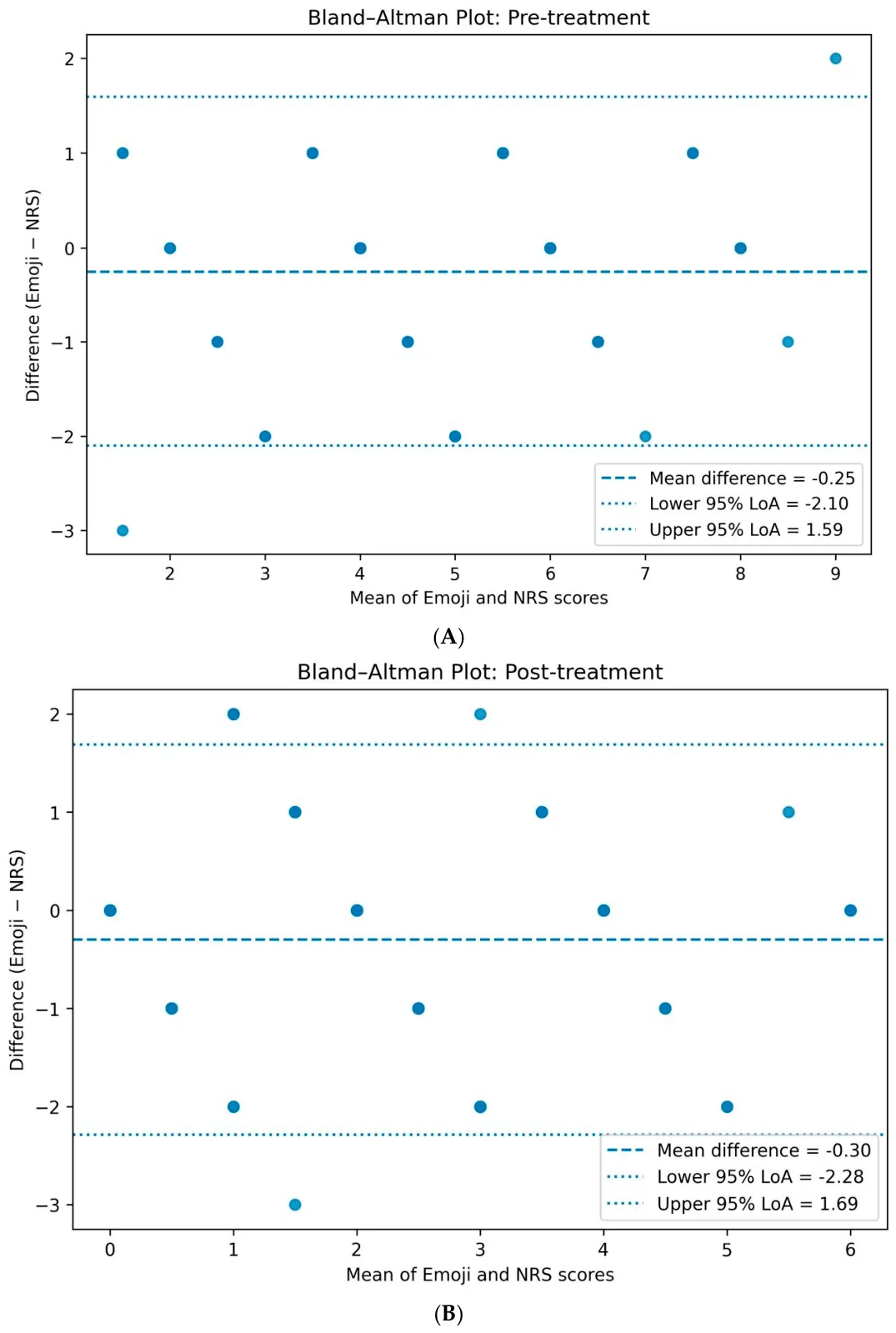
Bland–Altman analysis of agreement between the Emoji-Based Pain Scale and the Numeric Rating Scale. (**A**) Pre-treatment assessment, n = 87 paired observations. Mean paired difference: −0.25 points; 95% limits of agreement: −2.10 to 1.59. (**B**) Post-treatment assessment, n = 87 paired observations. Mean paired difference: −0.30 points; 95% limits of agreement: −2.28 to 1.69.

A strong positive monotonic association was observed between the Emoji-Based Pain Scale and the NRS at both assessment timepoints. Before analgesic treatment, Spearman’s correlation coefficient was ρ = 0.860 (*p* < 0.001). After treatment, the association remained strong, with ρ = 0.805 (*p* < 0.001). In the pre-treatment Bland–Altman analysis, the mean paired difference, calculated as the emoji score minus the NRS score, was −0.25 points. The 95% limits of agreement ranged from −2.10 to 1.59 points. In the post-treatment analysis, the mean paired difference was −0.30 points, with 95% limits of agreement ranging from −2.28 to 1.69 points. These findings indicate limited average systematic differences, with the Emoji-Based Pain Scale yielding scores approximately 0.25–0.30 points lower than the NRS. At the individual level, however, paired scores could differ by approximately two points. The instruments should therefore not be considered fully interchangeable. Part of this individual-level difference is attributable to scale granularity. The Emoji-Based Pain Scale permits only six even-numbered scores, whereas the NRS permits every integer between 0 and 10. Differences of approximately one point may therefore reflect quantization rather than clinically meaningful disagreement. Ordinal agreement between the Emoji-Based Pain Scale and the collapsed NRS categories was good at both assessment timepoints. Before treatment, quadratic-weighted Cohen’s kappa was 0.747 (95% bootstrap CI, 0.661–0.818). Exact categorical agreement was observed in 54 of 87 assessments (62.1%). After treatment, quadratic-weighted Cohen’s kappa was 0.720 (95% bootstrap CI, 0.609–0.806). Exact categorical agreement was observed in 51 of 87 assessments (58.6%). The weighted-kappa findings support good ordinal agreement while remaining consistent with the Bland–Altman conclusion that the instruments are not fully interchangeable (Figure 4).

In each panel, the central dashed line represents the mean paired difference, whereas the upper and lower dotted lines represent the 95% limits of agreement. Slight jitter and marker sizes proportional to the frequency of overlapping observations are used to display point density. The limited number of visually distinct coordinates reflects the discrete response formats and the resulting overlap of multiple observations.

The category mapping shown in Table 3 was used for the quadratic-weighted kappa analysis and should not be interpreted as an independently validated clinical conversion system.

### 3.4. Ancillary Rank-Ordering Exercise

The ancillary exercise included 50 adult volunteers. Their mean age was 51.6 ± 21.0 years, with a median age of 51 years and a range of 19–84 years. Twenty-seven participants were female and 23 were male. Twenty-eight participants were healthcare professionals or healthcare students, and 24 reported frequent emoji use.

All 50 participants independently reproduced the prespecified sequence:

U+1F600 → U+1F610 → U+1F641 → U+1F61F → U+1F623 → U+1F629.

Exact complete agreement was therefore observed in 50/50 participants (100%). No participant inverted U+1F623 and U+1F629. Overall interparticipant concordance was complete (Kendall’s W = 1.000). These findings support the intended ordinal sequence of the selected emojis in this ancillary sample. They do not, however, constitute comprehensive psychometric validation of the scale.

## 4. Discussion

This prospective pilot study found that a six-category Emoji-Based Pain Scale was feasible for use in a selected population of adult ED patients with clinically relevant language barriers. Almost 90% of participants completed the emoji scale without assistance, compared with approximately 70% for the NRS. The strong monotonic associations observed before and after analgesic treatment indicate that the ordered emoji responses tracked NRS scores at the group level. This interpretation was reinforced by the quadratic-weighted kappa analysis, which showed good ordinal agreement both before and after treatment. The weighted kappa values of 0.747 and 0.720 indicate that disagreements were generally limited in magnitude after accounting for the ordered structure of the categories. Exact categorical agreement, however, was observed in 62.1% of pre-treatment assessments and 58.6% of post-treatment assessments. The instruments therefore showed meaningful ordinal consistency but were not identical at the level of individual category assignment. Exploratory Bland–Altman analyses similarly showed limited average systematic differences, but individual paired scores could differ by approximately two points. Taken together, Spearman’s correlation, weighted kappa, and Bland–Altman analysis provide complementary information. Spearman’s coefficient describes monotonic association, weighted kappa evaluates agreement across ordered categories, and Bland–Altman analysis describes the magnitude and distribution of numerical differences. None of these analyses, either individually or in combination, establishes complete interchangeability. The different granularity of the instruments should be considered when interpreting the observed discrepancies. The emoji scale permits only six values, all of which are even numbers, whereas the NRS permits 11 integer responses. A one-point discrepancy may therefore arise even when the patient’s conceptual pain category is broadly similar across instruments. The parallel reduction in mean scores following analgesic treatment suggests that the emoji scale may reflect clinical change. Responsiveness was not formally evaluated, however, and the observed change cannot by itself establish sensitivity, validity, or reliability. A central methodological question concerned whether the selected emojis conveyed an intrinsic pain-intensity sequence independently of their ordered presentation. This issue could not be resolved by the original clinical study because the emojis were displayed horizontally in ascending order and associated with numerical values. The ancillary exercise therefore presented the same glyphs without labels and in a randomized order. All 50 participants reconstructed the intended sequence, including the two upper anchors. Overall interparticipant concordance was complete. This finding supports the face-validity of the selected ordering within the ancillary sample. Nevertheless, the exercise involved a convenience sample and did not constitute comprehensive cross-cultural validation. Complete agreement in this sample does not exclude different interpretations in other linguistic, cultural, socioeconomic, or age groups. Previous research provides relevant context. He et al. compared an emoji-based visual analogue pain scale with an NRS and reported substantial agreement in patients from a single hospital [15]. Subsequent correspondence highlighted methodological considerations regarding the interpretation and comparison of emoji-based pain instruments [16,17]. Li et al. undertook a more extensive development and validation process for an Emoji Faces Pain Scale in adult surgical patients, including evaluation on mobile devices and direct comparison with the Wong–Baker FACES Pain Rating Scale, Faces Pain Scale–Revised, NRS, and VAS [25]. Emojis have also been explored for emotion and mood assessment in other healthcare settings [18,19]. The present investigation differs from these studies in three principal respects. First, it focused specifically on ED patients with a clinically relevant language barrier. Second, it evaluated feasibility before and after analgesic treatment. Third, it used a fixed printed rendering rather than a mobile-device interface. At the same time, unlike Li et al., the present study did not include direct comparison with an established facial pain scale and should therefore be considered an exploratory pilot rather than a definitive validation study. Emojis are widely familiar, but they should not be described as universally interpreted or culturally neutral [20,21]. Their meaning may be affected by culture, familiarity, age, digital experience, and rendering. A relatively non-specific facial design may reduce some demographic cues present in realistic human faces, but freedom from age, gender, ethnic, or cultural bias cannot be assumed. The fixed printed rendering used in this study prevented variation among devices during data collection. Future digital use would require platform-specific testing because Unicode assigns code points and semantic descriptors but does not impose identical vendor-specific artwork [14,26,27,28,29,30,31].

### 4.1. Clinical Implications

The principal potential value of the Emoji-Based Pain Scale lies in its simplicity and accessibility. It may provide an additional means of eliciting a patient’s subjective pain report when numerical or verbal formats are difficult to use [20,21]. The higher rate of independent completion observed with the emoji scale suggests a practical usability advantage in this selected cohort. The good ordinal agreement with the NRS further supports its potential as a communication aid. Previous studies have likewise explored emoji-based tools for pain, mood, and emotional communication in medical settings [18,27]. This does not imply that the instrument should replace the NRS or an established visual pain scale. Rather, it may serve as a supplementary tool pending further validation. The scale should not be used in isolation to guide treatment. Pain management requires integration of the patient’s report with clinical examination, physiological findings, mechanism of illness or injury, previous treatment, and serial reassessment [4].

### 4.2. Medico-Legal and Ethical Implications

Italian Law No. 38/2010 requires pain intensity and its evolution during treatment to be documented in the medical record [3]. Language barriers do not remove this obligation, but they may make it more difficult to fulfil. A structured visual communication tool may help document that reasonable efforts were made to obtain the patient’s subjective pain report despite linguistic discordance. Such documentation may support continuity of care and demonstrate appropriate attention to the patient’s symptom experience within broader Italian patient-safety frameworks [22,23]. Professional responsibility in the Italian healthcare system also emphasizes appropriate documentation and adherence to accepted clinical practices [24]. The Emoji-Based Pain Scale has not, however, received specific legal recognition and has not yet undergone complete psychometric validation. No independent evidentiary, defensive, or protective legal value should therefore be attributed to its use. Pain assessment must also be distinguished from informed consent. An emoji pain scale cannot establish that a patient has understood the nature, risks, potential benefits, and alternatives of a proposed intervention. It cannot replace professional interpretation, cultural mediation, or the broader dialogue required for valid informed consent under Italian Law No. 219/2017 [27,30,31,32]. From an ethical perspective, efforts to provide accessible symptom-assessment tools are consistent with beneficence, justice, respect for autonomy, and non-discrimination. Their use should nevertheless remain proportionate to the available evidence and should not create a false impression of precision or legal validation.

### 4.3. Future Perspectives

Future studies should compare the Emoji-Based Pain Scale directly with established visual facial scales, including the Wong–Baker FACES Pain Rating Scale and Faces Pain Scale–Revised [15,33,34,35]. Formal psychometric evaluation should include construct validity, test–retest reliability when clinically appropriate, responsiveness, measurement error, cross-cultural interpretation, and clinically relevant thresholds [8,14]. Multicenter studies should enrol larger and more diverse populations and examine performance across age groups, levels of literacy and numeracy, cultural backgrounds, and clinical conditions. Patients with cognitive impairment should be evaluated in specifically designed studies using appropriate methodological and ethical safeguards [12]. The present findings should not be extrapolated to this population. Digital implementation may facilitate structured recording and longitudinal evaluation of pain scores. Such applications remain speculative and require independent assessment. Digital versions would also require testing of vendor-specific rendering and should not be assumed to be equivalent to the fixed printed scale used in the present study [16,19].

## 5. Conclusions

In this single-center prospective pilot study, a six-category Emoji-Based Pain Scale was feasible for use in adult ED patients with language barriers and was more frequently completed without assistance than the NRS. Emoji scores showed strong monotonic associations and good ordinal agreement with the NRS before and after analgesic treatment. Mean systematic differences were limited, but individual-level disagreement and different scale granularity indicate that the instruments should not be considered fully interchangeable. A post hoc ancillary ordering exercise showed complete agreement among 50 adult volunteers regarding the prespecified sequence of the six selected emojis. This finding supports the intended ordering in the tested sample but does not replace formal psychometric or cross-cultural validation. The Emoji-Based Pain Scale should therefore be regarded as a promising visual communication aid rather than a validated substitute for established pain-assessment instruments. Larger multicenter studies and direct comparison with established visual pain scales are required before routine clinical integration.

## 6. Limitations

This study has several limitations.

First, it was conducted at a single center and included a relatively small sample, limiting generalizability to other ED settings and sociocultural populations. Second, although potentially eligible patients were assessed consecutively during periods of investigator availability, uninterrupted screening was not performed. A complete screening log was not maintained, and the total number of potentially eligible patients and exclusions cannot be reported. Residual selection bias is therefore possible. Third, inclusion required sufficient communication through English, French, a bilingual healthcare professional, or an available cultural mediator. Patients for whom no working vehicular language or mediation support was available could not be enrolled. The study therefore excluded the most communication-disadvantaged patients and may have selected a comparatively more accessible subgroup. Fourth, patients with cognitive impairment or altered mental status were excluded because the protocol required reliable independent self-report. The findings cannot be generalized to cognitively impaired populations. Fifth, the Emoji-Based Pain Scale was always administered before the NRS. The administration order was not randomized, and an anchoring or carry-over effect cannot be excluded. The professionals administering the instruments were not blinded to the alternate score. Sixth, communication was conducted through different vehicular languages and, when necessary, different bilingual professionals or mediators. Although this reflects routine ED practice, variation in the explanations provided may have introduced interobserver variability. Seventh, the Emoji-Based Pain Scale contains six ordered categories assigned evenly spaced numerical values. Treating those values as an interval proxy in Bland–Altman analysis was exploratory and does not establish measurement equivalence. Eighth, the NRS and Emoji-Based Pain Scale have different response granularities. Some observed disagreement may therefore represent quantization rather than a clinically meaningful difference. Ninth, the weighted kappa analysis required collapsing the 11-point NRS into six prespecified bands. Although this approach permitted ordinal comparison with the six-category emoji scale, the thresholds were derived from the study’s conceptual mapping and have not been independently validated as clinical equivalence bands. Tenth, no patient selected the maximum emoji category in the clinical study. The upper anchor was therefore not evaluated directly in the patient-level association and agreement analyses. Eleventh, although the ancillary ordering exercise showed complete agreement, it was conducted post hoc in a convenience sample of 50 adults and was not a formal, prospectively designed cross-cultural or psychometric validation study. Finally, a single fixed printed rendering was used. Although this approach ensured consistency within the study, the findings cannot automatically be generalized to emojis rendered on other devices, operating systems, or software platforms.

Future multicenter studies with larger, multilingual and multicultural samples, direct comparison with established visual pain scales, and formal psychometric evaluation are required.

## Figures and Tables

**Table 1:** **Numeric Rating Scale (NRS).** The Numeric Rating Scale (NRS) is a unidimensional tool used to assess the intensity of pain. Patients are asked to rate their pain on a scale from 0 to 10, where 0 indicates no pain and 10 represents the worst imaginable pain.

Score	Pain Intensity Description
0	No pain
1	Minimal pain
2	Mild pain
3	Mild to moderate pain
4	Moderate pain
5	Moderate to severe pain
6	Severe pain
7	Very severe pain
8	Intense pain
9	Very intense pain
10	Worst imaginable pain

**Table 2:** Pain score comparison: Emoji vs. NRS.

Timepoint	Emoji Scale (Mean ± SD)	NRS (Mean ± SD)
Pre-treatment	4.9 ± 1.77	5.15 ± 1.65
Post-treatment	2.16 ± 1.62	2.46 ± 1.73

**Table 3:** Prespecified mapping of Emoji-Based Pain Scale categories to NRS score bands.

Emoji	Unicode	Scale Label	Emoji Score	Corresponding NRS Band
😀	U+1F600	No pain	0	0
😐	U+1F610	Mild pain	2	1–2
🙁	U+1F641	Moderate pain	4	3–4
😟	U+1F61F	Severe pain	6	5–6
😣	U+1F623	Very severe pain	8	7–8
😩	U+1F629	Worst possible pain	10	9–10

## Data Availability

The de-identified data supporting the findings of this study are available from the corresponding author upon reasonable request, subject to applicable ethical and institutional requirements.

## References

[1] IASP (2020). Announces Revised Definition of Pain.

[2] Raja S.N., Carr D.B., Cohen M., Finnerup N.B., Flor H., Gibson S., Keefe F.J., Mogil J.S., Ringkamp M., Sluka K.A. (2020). The Revised International Association for the Study of Pain Definition of Pain: Concepts, Challenges, and Compromises. Pain.

[3] Gazzetta Ufficiale. https://www.gazzettaufficiale.it/gunewsletter/dettaglio.jsp?service=1&datagu=2010-03-19&task=dettaglio&numgu=65&redaz=010G0056&tmstp=1269600292070.

[4] WHO Revision of Pain Management Guidelines. https://www.who.int/who-revision-of-pain-management-guidelines.

[5] Gensini G.F. (2011). New Disposition for Pain Therapy in Italy, Center for Headache Should Be Integrated in the Network. Neurol. Sci..

[6] Ripamonti C.I., Bandieri E., Roila F., ESMO Guidelines Working Group (2011). Management of Cancer Pain: ESMO Clinical Practice Guidelines. Ann. Oncol..

[7] Nimmaanrat S., Thepsuwan A., Tipchatyotin S., Jensen M.P. (2024). Measuring Pain Intensity in Older Patients: A Comparison of Five Scales. BMC Geriatr..

[8] Atisook R., Euasobhon P., Saengsanon A., Jensen M.P. (2021). Validity and Utility of Four Pain Intensity Measures for Use in International Research. J. Pain Res..

[9] Jensen M.P., Castarlenas E., Roy R., Tomé Pires C., Racine M., Pathak A., Miró J. (2019). The Utility and Construct Validity of Four Measures of Pain Intensity: Results from a University-Based Study in Spain. Pain Med..

[10] Hjermstad M.J., Fayers P.M., Haugen D.F., Caraceni A., Hanks G.W., Loge J.H., Fainsinger R., Aass N., Kaasa S., European Palliative Care Research Collaborative (EPCRC) (2011). Studies Comparing Numerical Rating Scales, Verbal Rating Scales, and Visual Analogue Scales for Assessment of Pain Intensity in Adults: A Systematic Literature Review. J. Pain Symptom Manag..

[11] Boonstra A.M., Schiphorst Preuper H.R., Reneman M.F., Posthumus J.B., Stewart R.E. (2008). Reliability and Validity of the Visual Analogue Scale for Disability in Patients with Chronic Musculoskeletal Pain. Int. J. Rehabil. Res..

[12] Herr K., Coyne P.J., Key T., Manworren R., McCaffery M., Merkel S., Pelosi-Kelly J., Wild L., American Society for Pain Management Nursing (2006). Pain Assessment in the Nonverbal Patient: Position Statement with Clinical Practice Recommendations. Pain Manag. Nurs..

[13] Couper M.P., Tourangeau R., Conrad F.G., Singer E. (2006). Evaluating the Effectiveness of Visual Analog Scales: A Web Experiment. Soc. Sci. Comput. Rev..

[14] Chien C.-W., Bagraith K.S., Khan A., Deen M., Strong J. (2013). Comparative Responsiveness of Verbal and Numerical Rating Scales to Measure Pain Intensity in Patients with Chronic Pain. J. Pain.

[15] Wong D.L., Baker C.M. (1988). Pain in Children: Comparison of Assessment Scales. Pediatr. Nurs..

[16] Full Emoji List, V16.0. https://unicode.org/emoji/charts/full-emoji-list.html.

[17] Diana Unicode—The World Standard for Text and Emoji. https://home.unicode.org/.

[18] He S., Renne A., Argandykov D., Convissar D., Lee J. (2022). Comparison of an Emoji-Based Visual Analog Scale With a Numeric Rating Scale for Pain Assessment. J. Am. Med. Assoc..

[19] Unicode & The Unicode Standard. https://emojipedia.org/the-unicode-consortium.

[20] MedXCom (2023). Effective Communication in a Multicultural Medical Setting. MedXCom. https://medx.com/multicultural-medical-setting/.

[21] Snyder J. The Picture of Health: Visual Representation as Communicative Practice in Healthcare Settings. https://ics.uci.edu/~yunanc/cscw2013health/Final%20submissions/Snyder_The%20Picture%20of%20Health%20-%20Visual%20Representation%20as%20Communicative%20Practice%20in%20Healthcare%20Settings_Final.pdf#1#1.

[22] Huber M., Stamer U. (2025). Pain Assessment on a Numerical Scale with Uncertainty Intervals: A Proof-of-Concept Simulation Study. Front. Pain Res..

[23] King M., Patterson E.D. (2023). The FACES of the Future: Emojis in Perioperative Medicine. Anesth. Analg..

[24] Renne A., He S., Lee J. (2022). An Emoji-Based Visual Analog Scale Compared With a Numeric Rating Scale for Pain Assessment-Reply. J. Am. Med. Assoc..

[25] Moisset X., Attal N., Ciampi de Andrade D. (2022). An Emoji-Based Visual Analog Scale Compared With a Numeric Rating Scale for Pain Assessment. J. Am. Med. Assoc..

[26] Marañón-Vásquez G.A., Maia L.C., Barreto L.S.d.C., da Cruz M.F., Jural L.A., Araújo M.T.d.S., Pithon M.M. (2022). Emoji as Promising Tools for Emotional Evaluation in Orthodontics. Prog. Orthod..

[27] Pourmand A., Quan T., Amini S.B., Sikka N. (2020). Can Emojis Assess Patients’ Mood and Emotion in the Emergency Department? An Emoji-Based Study. Am. J. Emerg. Med..

[28] Lai D., Lee J.8., He S. (2021). Emoji for the Medical Community-Challenges and Opportunities. J. Am. Med. Assoc..

[29] Troiano G., Nante N. (2018). Emoji: What Does the Scientific Literature Say about Them? A New Way to Communicate in the 21st Century. J. Hum. Behav. Soc. Environ..

[30] Neesu K., Saxena A., Chauhan S.P., Verma N. (2025). Rationality of Efficacy of the Treatment Modalities-Modified Atraumatic Restorative Treatment and Hall Technique by Comparing Clinical and Radiographic Parameters along with Posttreatment Behavioral Assessment of Patients through Face Pain Scale-Revised: An In Vivo Study. Int. J. Clin. Pediatr. Dent..

[31] Martines V., Magnaldi S., Di Fazio N., Volonnino G., Marinelli E., De Paola L. (2025). Enhancing Patient Safety and Delineating Professional Responsibility in Radiological Procedures Involving Contrast Medium Administration. Clin. Ter..

[32] Montanari Vergallo G., Zaami S. (2018). Guidelines and Best Practices: Remarks on the Gelli-Bianco Law. Clin. Ter..

[33] Albolino S., Bellandi T., Cappelletti S., Di Paolo M., Fineschi V., Frati P., Offidani C., Tanzini M., Tartaglia R., Turillazzi E. (2019). New Rules on Patient’s Safety and Professional Liability for the Italian Health Service. Curr. Pharm. Biotechnol..

[34] Li L., Wu S., Wang J., Wang C., Zuo W., Yu L., Song J. (2023). Development of the Emoji Faces Pain Scale and Its Validation on Mobile Devices in Adult Surgery Patients: Longitudinal Observational Study. J. Med. Internet Res..

[35] Cirillo B., Tarallo M., Duranti G., Sapienza P., Cicerchia P.M., Simonelli L., Cirocchi R., Matteucci M., Mingoli A., Brachini G. (2025). Electric Scooter Trauma in Rome: A Three-Year Analysis from a Tertiary Care Hospital. J. Clin. Med..

